# Bis[*N*′-(2-oxo-1*H*-indol-3-ylidene)thiophene-2-carbohydrazidato-κ^3^ *O*,*N*′,*O*′]zinc(II) *N*,*N*-dimethyl­formide mono­solvate monohydrate

**DOI:** 10.1107/S1600536810039504

**Published:** 2010-10-23

**Authors:** Siti Nadiah Abdul Halim, Hapipah Mohd Ali, Seik Weng Ng

**Affiliations:** a Department of Chemistry, University of Malaya, 50603 Kuala Lumpur, Malaysia

## Abstract

The metal atom of the title compound, [Zn(C_13_H_8_N_3_O_2_S)_2_]·C_3_H_7_NO·H_2_O, is *O*,*N*,*O*′-chelated by two deprotonated Schiff bases and it exists in a distorted octa­hedral geometry. The N–H groups of the ligands, the carbonyl group of the DMF mol­ecule and uncoordinated water mol­ecule engage in N—H⋯O and O—H⋯O inter­actions, generating a hydrogen-bonded ribbon that propagates along [110]. One thienyl ring is disordered over two positions in a 1:1 ratio.

## Related literature

For the crystal structure of [Zn(C_13_H_8_N_3_O_2_S)_2_]·1.75CH_3_OH, see: Rodríguez-Argüelles *et al.* (2009 ▶).
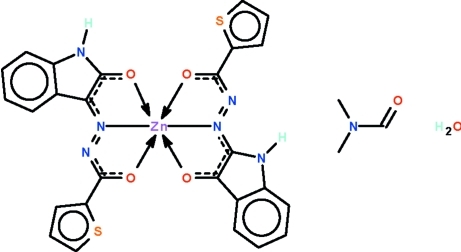


## Experimental

### 

#### Crystal data

[Zn(C_13_H_8_N_3_O_2_S)_2_]·C_3_H_7_NO·H_2_O
*M* *_r_* = 697.05
Triclinic, 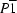

*a* = 11.5250 (8) Å
*b* = 12.0656 (8) Å
*c* = 13.4643 (9) Å
α = 105.913 (1)°
β = 100.531 (1)°
γ = 114.754 (1)°
*V* = 1537.25 (18) Å^3^
*Z* = 2
Mo *K*α radiation
μ = 0.99 mm^−1^
*T* = 293 K
0.22 × 0.16 × 0.08 mm

#### Data collection

Bruker SMART area-detector diffractometer
Absorption correction: multi-scan (*SADABS*; Sheldrick, 1996 ▶) *T*_min_ = 0.812, *T*_max_ = 0.925
13275 measured reflections
6630 independent reflections
3036 reflections with *I* > 2σ(*I*)
*R*_int_ = 0.044

#### Refinement

*R*[*F* ^2^ > 2σ(*F* ^2^)] = 0.059
*wR*(*F* ^2^) = 0.182
*S* = 0.98
6630 reflections
432 parameters
77 restraints
H atoms treated by a mixture of independent and constrained refinement
Δρ_max_ = 0.56 e Å^−3^
Δρ_min_ = −0.35 e Å^−3^

### 

Data collection: *SMART* (Bruker, 2001 ▶); cell refinement: *SAINT* (Bruker, 2001 ▶); data reduction: *SAINT*; program(s) used to solve structure: *SHELXS97* (Sheldrick, 2008 ▶); program(s) used to refine structure: *SHELXL97* (Sheldrick, 2008 ▶); molecular graphics: *X-SEED* (Barbour, 2001 ▶); software used to prepare material for publication: *publCIF* (Westrip, 2010 ▶).

## Supplementary Material

Crystal structure: contains datablocks global, I. DOI: 10.1107/S1600536810039504/ci5163sup1.cif

Structure factors: contains datablocks I. DOI: 10.1107/S1600536810039504/ci5163Isup2.hkl

Additional supplementary materials:  crystallographic information; 3D view; checkCIF report

## Figures and Tables

**Table 1:** Selected bond lengths (Å)

Zn1—O1	2.103 (3)
Zn1—O2	2.370 (3)
Zn1—O3	2.043 (3)
Zn1—O4	2.440 (3)
Zn1—N2	2.017 (4)
Zn1—N5	2.022 (4)

**Table 2:** Hydrogen-bond geometry (Å, °)

*D*—H⋯*A*	*D*—H	H⋯*A*	*D*⋯*A*	*D*—H⋯*A*
O1*W*—H1w1⋯O2	0.84 (1)	2.06 (3)	2.859 (5)	158 (6)
O1*W*—H1w2⋯O5	0.84 (1)	2.31 (8)	2.771 (9)	115 (7)
N3—H3*N*⋯O1*W*^i^	0.84 (1)	1.98 (1)	2.813 (6)	173 (5)
N6—H6*N*⋯O4^ii^	0.84 (1)	2.06 (2)	2.884 (5)	169 (5)

Symmetry codes: (i) 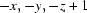; (ii) 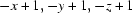.

**Fig. 1.**
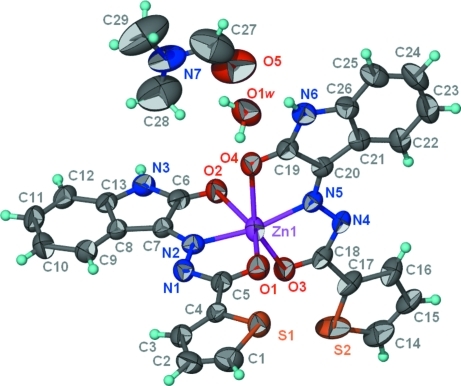
Displacement ellipsoid plot (Barbour, 2001) of [Zn(C13H8N3O2S)2].C3H7NO.H2O at the 50% probability level; H atoms are drawn as spheres of arbitrary radius. The disorder in the thienyl ring is not shown.

## supplementary crystallographic information

### Comment

Divalent cobalt, nickel, copper and zinc derivatives of Schiff base condensation product of isatin and 2-thienylcarboxylic acid hydrazide have been synthesized, and these are reported along with the crystal structure of the zinc derivative, which crystallizes with 1.75 molecules of methanol. The geometry is described as tetrahedral; however, if two weaker Zn···O interations are considered as bonding, the geometry is, in fact, octahedral (Rodríguez-Argüelles *et al.*, 2009). The other compounds are expected to be chelated by the deprotonated Schiff base in a terdentate manner; for it to chelate, the anion has to rotate about the nitrogen-nitrogen bond. Such a rotation is observed in the present zinc complex, which crystallizes from DMF as a monohydrated monosolvate (Scheme I). The divalent metal atom is *O*,*N*,*O*'-chelated by two deprotonated Schiff base in an octahedral geometry (Fig. 1). The amino group, the carbonyl group of the DMF and the lattice water molecule engage in N–H···O and O–H···O hydrogen bonding interactions to generate a hydrogen-bonded ribbon that propagates along [110]. One thienyl ring is disordered over two positions in a 1:1 ratio.

### Experimental

The Schiff base was synthesized by condensing isatin and furoylhydrazine according to a literature procedure (Rodríguez-Argüelles *et al.*, 2009). Zinc acetate (1 mmol) and the Schiff base (2 mmol) were heated in ethanol (100 ml) for 5 h; several drops of triethylamine were added. The solvent was removed and the product purified by recrystallization from DMF.

### Refinement

One thienyl ring is disordered over two positions. The disorder was modelled as follows: the C—C distances were restrained to 1.42 (1) Å and the C–S ones to 1.72 (1) Å. The displacement parameters of atoms S2 were paired with those of C16' (S2' with C16), and those of C14 with those of C15' (C14' with C15). The anisotropic displacement parameters were restrained to be nearly isotropic. The five atoms of each component ring were restrained to lie on a plane. As the disorder refined to a 1:1 ratio, the occupancy for each component was finally fixed as 0.50.

For the DMF molecule, the C—O distance was restrained to 1.25 (1) Å, the C_carbonyl_—N distance to 1.35 (1) Å and the C_methyl_—N distance to 1.45 (1) %A. The displacement parameters were also restrained to be nearly isotropic.

Carbon-bound H-atoms were placed in calculated positions (C–H = 0.93–0.96 Å) and were included in the refinement in the riding model approximation, with *U_iso_*(H) set to 1.2 to 1.5*U_eq_*(C). The amino and water H-atoms were located in a difference Fourier map, and were refined with a N–H/O–H distance restraint of 0.84 (1) Å; their U_iso_ parameters were freely refined. For the water molecule, the H···H distance was restrained to 1.37 (1) Å.

### Crystal data

**Table:**

[Zn(C_13_H_8_N_3_O_2_S)_2_]·C_3_H_7_NO·H_2_O	*Z* = 2
*M**_r_* = 697.05	*F*(000) = 716
Triclinic, *P*1	*D*_x_ = 1.506 Mg m^−^^3^
Hall symbol: -P 1	Mo *K*α radiation, λ = 0.71073 Å
*a* = 11.5250 (8) Å	Cell parameters from 2596 reflections
*b* = 12.0656 (8) Å	θ = 2.6–21.1°
*c* = 13.4643 (9) Å	µ = 0.99 mm^−^^1^
α = 105.913 (1)°	*T* = 293 K
β = 100.531 (1)°	Plate, yellow
γ = 114.754 (1)°	0.22 × 0.16 × 0.08 mm
*V* = 1537.25 (18) Å^3^	

### Data collection

**Table:**

Bruker SMART area-detector diffractometer	6630 independent reflections
Radiation source: fine-focus sealed tube	3036 reflections with *I* > 2σ(*I*)
graphite	*R*_int_ = 0.044
ω scans	θ_max_ = 27.0°, θ_min_ = 1.7°
Absorption correction: multi-scan (*SADABS*; Sheldrick, 1996)	*h* = −14→14
*T*_min_ = 0.812, *T*_max_ = 0.925	*k* = −15→15
13275 measured reflections	*l* = −17→17

### Refinement

**Table:**

Refinement on *F*^2^	Primary atom site location: structure-invariant direct methods
Least-squares matrix: full	Secondary atom site location: difference Fourier map
*R*[*F*^2^ > 2σ(*F*^2^)] = 0.059	Hydrogen site location: inferred from neighbouring sites
*wR*(*F*^2^) = 0.182	H atoms treated by a mixture of independent and constrained refinement
*S* = 0.98	*w* = 1/[σ^2^(*F*_o_^2^) + (0.0886*P*)^2^] where *P* = (*F*_o_^2^ + 2*F*_c_^2^)/3
6630 reflections	(Δ/σ)_max_ = 0.001
432 parameters	Δρ_max_ = 0.56 e Å^−^^3^
77 restraints	Δρ_min_ = −0.35 e Å^−^^3^

### Fractional atomic coordinates and isotropic or equivalent isotropic displacement parameters (Å^2^)

**Table:**

	*x*	*y*	*z*	*U*_iso_*/*U*_eq_	Occ. (<1)
Zn1	0.50562 (6)	0.34927 (6)	0.74919 (5)	0.0638 (3)	
S1	0.91009 (13)	0.79052 (15)	1.01272 (12)	0.0733 (4)	
S2	0.6067 (6)	0.0742 (6)	0.9286 (3)	0.1011 (11)	0.50
C14	0.6512 (13)	−0.0461 (12)	0.9100 (13)	0.100 (4)	0.50
H14	0.6557	−0.0852	0.9602	0.120*	0.50
C15	0.6802 (16)	−0.0815 (17)	0.8139 (11)	0.079 (3)	0.50
H15	0.7059	−0.1449	0.7903	0.095*	0.50
C16	0.6622 (12)	−0.0003 (13)	0.7586 (16)	0.0770 (8)	0.50
H16	0.6759	−0.0058	0.6919	0.092*	0.50
S2'	0.6837 (4)	−0.0034 (4)	0.7590 (5)	0.0770 (8)	0.50
C14'	0.6977 (14)	−0.0544 (14)	0.8662 (9)	0.079 (3)	0.50
H14'	0.7307	−0.1121	0.8699	0.095*	0.50
C15'	0.6541 (17)	0.0001 (16)	0.9468 (15)	0.100 (4)	0.50
H15'	0.6533	−0.0146	1.0110	0.120*	0.50
C16'	0.6114 (16)	0.0819 (19)	0.9129 (10)	0.1011 (11)	0.50
H16'	0.5783	0.1286	0.9546	0.121*	0.50
O1	0.6775 (3)	0.5329 (3)	0.8550 (3)	0.0683 (10)	
O2	0.2678 (3)	0.2050 (3)	0.6643 (3)	0.0664 (10)	
O3	0.5375 (4)	0.2324 (4)	0.8228 (3)	0.0671 (10)	
O4	0.4945 (4)	0.4247 (4)	0.5989 (3)	0.0678 (10)	
O5	0.2073 (8)	0.2034 (7)	0.3516 (6)	0.171 (3)	
O1W	0.1468 (5)	0.0147 (4)	0.4424 (3)	0.0953 (13)	
H1W1	0.184 (7)	0.054 (6)	0.5112 (12)	0.143*	
H1W2	0.101 (7)	0.048 (7)	0.421 (5)	0.143*	
N1	0.5088 (4)	0.5749 (4)	0.9029 (3)	0.0581 (10)	
N2	0.4269 (4)	0.4546 (4)	0.8249 (3)	0.0560 (10)	
N3	0.0860 (4)	0.2331 (4)	0.6887 (3)	0.0613 (11)	
H3N	0.014 (3)	0.163 (3)	0.648 (3)	0.074*	
N4	0.6067 (4)	0.1698 (4)	0.6741 (3)	0.0502 (10)	
N5	0.5693 (4)	0.2512 (4)	0.6455 (3)	0.0511 (10)	
N6	0.5631 (5)	0.3803 (5)	0.4471 (4)	0.0640 (11)	
H6N	0.556 (5)	0.440 (4)	0.431 (4)	0.077*	
N7	0.1188 (7)	0.3387 (6)	0.3769 (6)	0.112 (2)	
C1	0.9569 (7)	0.9300 (6)	1.1235 (5)	0.0846 (18)	
H1	1.0461	0.9988	1.1607	0.102*	
C2	0.8526 (7)	0.9290 (6)	1.1500 (5)	0.0799 (17)	
H2	0.8608	0.9973	1.2089	0.096*	
C3	0.7278 (6)	0.8158 (6)	1.0816 (4)	0.0638 (14)	
H3B	0.6448	0.8003	1.0900	0.077*	
C4	0.7431 (5)	0.7305 (5)	1.0006 (4)	0.0515 (12)	
C5	0.6426 (5)	0.6058 (5)	0.9145 (4)	0.0548 (12)	
C6	0.2177 (5)	0.2683 (5)	0.7082 (4)	0.0575 (13)	
C7	0.2954 (5)	0.4045 (5)	0.7958 (4)	0.0524 (12)	
C8	0.1993 (5)	0.4411 (5)	0.8260 (4)	0.0530 (12)	
C9	0.2121 (5)	0.5509 (5)	0.9032 (4)	0.0631 (14)	
H9	0.2972	0.6224	0.9490	0.076*	
C10	0.0925 (6)	0.5518 (6)	0.9109 (5)	0.0765 (16)	
H10	0.0975	0.6246	0.9624	0.092*	
C11	−0.0324 (6)	0.4443 (7)	0.8419 (5)	0.0742 (16)	
H11	−0.1103	0.4473	0.8477	0.089*	
C12	−0.0470 (5)	0.3327 (6)	0.7647 (5)	0.0659 (15)	
H12	−0.1322	0.2607	0.7199	0.079*	
C13	0.0711 (5)	0.3333 (5)	0.7572 (4)	0.0553 (12)	
C17	0.6223 (5)	0.0882 (5)	0.8126 (4)	0.0690 (15)	
C18	0.5854 (4)	0.1691 (5)	0.7701 (4)	0.0543 (12)	
C19	0.5399 (5)	0.3688 (5)	0.5400 (4)	0.0553 (12)	
C20	0.5817 (4)	0.2732 (5)	0.5581 (4)	0.0505 (11)	
C21	0.6287 (5)	0.2316 (5)	0.4702 (4)	0.0542 (12)	
C22	0.6786 (5)	0.1459 (5)	0.4447 (4)	0.0648 (14)	
H22	0.6872	0.1001	0.4884	0.078*	
C23	0.7155 (6)	0.1303 (7)	0.3521 (5)	0.0864 (18)	
H23	0.7499	0.0733	0.3333	0.104*	
C24	0.7022 (7)	0.1980 (7)	0.2870 (5)	0.092 (2)	
H24	0.7275	0.1847	0.2249	0.111*	
C25	0.6524 (6)	0.2849 (6)	0.3109 (5)	0.0785 (17)	
H25	0.6436	0.3303	0.2668	0.094*	
C26	0.6166 (5)	0.3001 (5)	0.4041 (4)	0.0630 (14)	
C27	0.1813 (11)	0.2814 (11)	0.3207 (9)	0.178 (5)	
H27	0.2033	0.3002	0.2619	0.214*	
C28	0.1061 (10)	0.3139 (10)	0.4682 (8)	0.179 (4)	
H28A	0.1924	0.3332	0.5134	0.268*	
H28B	0.0408	0.2223	0.4468	0.268*	
H28C	0.0760	0.3689	0.5087	0.268*	
C29	0.0861 (11)	0.4222 (11)	0.3410 (10)	0.221 (5)	
H29A	0.0275	0.3750	0.2656	0.331*	
H29B	0.1677	0.4969	0.3474	0.331*	
H29C	0.0405	0.4527	0.3851	0.331*	

### Atomic displacement parameters (Å^2^)

**Table:**

	*U*^11^	*U*^22^	*U*^33^	*U*^12^	*U*^13^	*U*^23^
Zn1	0.0637 (4)	0.0688 (4)	0.0754 (5)	0.0417 (4)	0.0338 (3)	0.0303 (4)
S1	0.0478 (8)	0.0769 (10)	0.0803 (10)	0.0253 (8)	0.0194 (7)	0.0208 (8)
S2	0.139 (3)	0.104 (2)	0.073 (2)	0.057 (2)	0.0280 (18)	0.0614 (18)
C14	0.120 (6)	0.100 (9)	0.103 (8)	0.066 (5)	0.024 (5)	0.063 (7)
C15	0.078 (5)	0.083 (6)	0.091 (9)	0.049 (4)	0.012 (6)	0.049 (7)
C16	0.0690 (19)	0.0825 (17)	0.112 (2)	0.0505 (15)	0.0397 (17)	0.0564 (16)
S2'	0.0690 (19)	0.0825 (17)	0.112 (2)	0.0505 (15)	0.0397 (17)	0.0564 (16)
C14'	0.078 (5)	0.083 (6)	0.091 (9)	0.049 (4)	0.012 (6)	0.049 (7)
C15'	0.120 (6)	0.100 (9)	0.103 (8)	0.066 (5)	0.024 (5)	0.063 (7)
C16'	0.139 (3)	0.104 (2)	0.073 (2)	0.057 (2)	0.0280 (18)	0.0614 (18)
O1	0.055 (2)	0.066 (2)	0.076 (2)	0.026 (2)	0.0219 (19)	0.023 (2)
O2	0.055 (2)	0.072 (2)	0.067 (2)	0.030 (2)	0.0222 (18)	0.019 (2)
O3	0.076 (2)	0.082 (3)	0.070 (2)	0.050 (2)	0.038 (2)	0.041 (2)
O4	0.083 (3)	0.083 (3)	0.072 (2)	0.057 (2)	0.039 (2)	0.044 (2)
O5	0.216 (6)	0.118 (4)	0.149 (5)	0.074 (4)	0.018 (4)	0.052 (4)
O1W	0.092 (4)	0.075 (3)	0.076 (3)	0.028 (3)	0.016 (3)	0.002 (2)
N1	0.058 (3)	0.061 (3)	0.062 (3)	0.031 (2)	0.025 (2)	0.027 (2)
N2	0.056 (3)	0.061 (3)	0.052 (2)	0.027 (2)	0.018 (2)	0.028 (2)
N3	0.038 (3)	0.068 (3)	0.058 (3)	0.016 (2)	0.012 (2)	0.019 (2)
N4	0.040 (2)	0.054 (2)	0.064 (3)	0.026 (2)	0.019 (2)	0.030 (2)
N5	0.043 (2)	0.053 (2)	0.060 (3)	0.022 (2)	0.0171 (19)	0.027 (2)
N6	0.070 (3)	0.071 (3)	0.065 (3)	0.035 (3)	0.030 (2)	0.041 (3)
N7	0.107 (4)	0.096 (4)	0.106 (4)	0.029 (3)	0.013 (3)	0.052 (4)
C1	0.070 (4)	0.075 (4)	0.071 (4)	0.019 (3)	0.008 (3)	0.014 (3)
C2	0.092 (5)	0.085 (5)	0.056 (4)	0.045 (4)	0.021 (4)	0.018 (3)
C3	0.066 (4)	0.081 (4)	0.067 (3)	0.044 (3)	0.031 (3)	0.042 (3)
C4	0.054 (3)	0.059 (3)	0.052 (3)	0.033 (3)	0.018 (2)	0.028 (3)
C5	0.052 (3)	0.062 (3)	0.061 (3)	0.030 (3)	0.023 (3)	0.034 (3)
C6	0.048 (3)	0.071 (4)	0.052 (3)	0.025 (3)	0.018 (3)	0.029 (3)
C7	0.039 (3)	0.069 (3)	0.056 (3)	0.025 (3)	0.020 (2)	0.033 (3)
C8	0.042 (3)	0.068 (3)	0.054 (3)	0.026 (3)	0.019 (2)	0.029 (3)
C9	0.053 (3)	0.069 (4)	0.062 (3)	0.027 (3)	0.019 (3)	0.023 (3)
C10	0.077 (4)	0.085 (4)	0.081 (4)	0.048 (4)	0.040 (4)	0.032 (4)
C11	0.058 (4)	0.102 (5)	0.090 (4)	0.049 (4)	0.038 (3)	0.051 (4)
C12	0.040 (3)	0.084 (4)	0.077 (4)	0.026 (3)	0.021 (3)	0.044 (3)
C13	0.046 (3)	0.072 (4)	0.058 (3)	0.030 (3)	0.021 (3)	0.037 (3)
C17	0.050 (3)	0.072 (4)	0.091 (4)	0.028 (3)	0.017 (3)	0.048 (3)
C18	0.035 (3)	0.054 (3)	0.073 (4)	0.019 (2)	0.013 (2)	0.032 (3)
C19	0.051 (3)	0.062 (3)	0.061 (3)	0.027 (3)	0.022 (3)	0.035 (3)
C20	0.044 (3)	0.051 (3)	0.054 (3)	0.019 (2)	0.015 (2)	0.026 (2)
C21	0.046 (3)	0.048 (3)	0.062 (3)	0.017 (2)	0.019 (2)	0.022 (3)
C22	0.058 (3)	0.061 (3)	0.068 (4)	0.027 (3)	0.023 (3)	0.016 (3)
C23	0.073 (4)	0.087 (5)	0.093 (5)	0.040 (4)	0.040 (4)	0.019 (4)
C24	0.090 (5)	0.097 (5)	0.073 (4)	0.035 (4)	0.046 (4)	0.017 (4)
C25	0.077 (4)	0.076 (4)	0.064 (4)	0.019 (3)	0.032 (3)	0.026 (3)
C26	0.052 (3)	0.067 (4)	0.060 (3)	0.020 (3)	0.023 (3)	0.025 (3)
C27	0.180 (8)	0.159 (8)	0.140 (7)	0.033 (6)	0.068 (7)	0.055 (6)
C28	0.154 (7)	0.191 (8)	0.180 (7)	0.049 (6)	0.052 (6)	0.121 (7)
C29	0.174 (9)	0.197 (8)	0.265 (10)	0.071 (7)	−0.002 (6)	0.139 (8)

### Geometric parameters (Å, °)

**Table:**

Zn1—O1	2.103 (3)	N7—C28	1.360 (7)
Zn1—O2	2.370 (3)	N7—C27	1.378 (8)
Zn1—O3	2.043 (3)	N7—C29	1.382 (8)
Zn1—O4	2.440 (3)	C1—C2	1.311 (8)
Zn1—N2	2.017 (4)	C1—H1	0.93
Zn1—N5	2.022 (4)	C2—C3	1.402 (8)
S1—C4	1.705 (5)	C2—H2	0.93
S1—C1	1.708 (6)	C3—C4	1.373 (7)
S2—C17	1.650 (6)	C3—H3B	0.93
S2—C14	1.703 (9)	C4—C5	1.429 (7)
C14—C15	1.394 (10)	C6—C7	1.487 (7)
C14—H14	0.93	C7—C8	1.437 (6)
C15—C16	1.440 (10)	C8—C9	1.372 (7)
C15—H15	0.93	C8—C13	1.401 (7)
C16—C17	1.410 (10)	C9—C10	1.406 (7)
C16—H16	0.93	C9—H9	0.93
S2'—C17	1.627 (6)	C10—C11	1.380 (8)
S2'—C14'	1.724 (9)	C10—H10	0.93
C14'—C15'	1.395 (10)	C11—C12	1.378 (8)
C14'—H14'	0.93	C11—H11	0.93
C15'—C16'	1.413 (10)	C12—C13	1.381 (7)
C15'—H15'	0.93	C12—H12	0.93
C16'—C17	1.397 (10)	C17—C18	1.425 (6)
C16'—H16'	0.93	C19—C20	1.483 (6)
O1—C5	1.267 (5)	C20—C21	1.443 (6)
O2—C6	1.231 (6)	C21—C22	1.376 (7)
O3—C18	1.261 (5)	C21—C26	1.396 (7)
O4—C19	1.230 (5)	C22—C23	1.378 (7)
O5—C27	1.246 (8)	C22—H22	0.93
O1W—H1W1	0.842 (11)	C23—C24	1.381 (8)
O1W—H1W2	0.842 (11)	C23—H23	0.93
N1—N2	1.325 (5)	C24—C25	1.385 (9)
N1—C5	1.394 (6)	C24—H24	0.93
N2—C7	1.304 (6)	C25—C26	1.381 (7)
N3—C6	1.343 (6)	C25—H25	0.93
N3—C13	1.400 (7)	C27—H27	0.93
N3—H3N	0.837 (11)	C28—H28A	0.96
N4—N5	1.341 (5)	C28—H28B	0.96
N4—C18	1.361 (6)	C28—H28C	0.96
N5—C20	1.295 (6)	C29—H29A	0.96
N6—C19	1.357 (6)	C29—H29B	0.96
N6—C26	1.404 (7)	C29—H29C	0.96
N6—H6N	0.837 (11)		
			
N2—Zn1—N5	167.72 (15)	O2—C6—C7	125.4 (4)
N2—Zn1—O3	114.39 (14)	N3—C6—C7	105.6 (5)
N5—Zn1—O3	77.23 (14)	N2—C7—C8	138.3 (5)
N2—Zn1—O1	76.24 (16)	N2—C7—C6	113.8 (4)
N5—Zn1—O1	106.84 (14)	C8—C7—C6	107.9 (4)
O3—Zn1—O1	98.83 (15)	C9—C8—C13	121.1 (5)
N2—Zn1—O2	76.55 (15)	C9—C8—C7	133.6 (5)
N5—Zn1—O2	99.15 (14)	C13—C8—C7	105.4 (4)
O3—Zn1—O2	95.14 (14)	C8—C9—C10	117.8 (5)
O1—Zn1—O2	152.62 (13)	C8—C9—H9	121.1
N2—Zn1—O4	92.75 (13)	C10—C9—H9	121.1
N5—Zn1—O4	75.43 (14)	C11—C10—C9	119.9 (6)
O3—Zn1—O4	152.55 (13)	C11—C10—H10	120.1
O1—Zn1—O4	91.31 (13)	C9—C10—H10	120.1
O2—Zn1—O4	86.90 (12)	C12—C11—C10	123.0 (5)
C4—S1—C1	91.4 (3)	C12—C11—H11	118.5
C17—S2—C14	92.9 (8)	C10—C11—H11	118.5
C15—C14—S2	115.0 (15)	C11—C12—C13	116.7 (5)
C15—C14—H14	122.5	C11—C12—H12	121.6
S2—C14—H14	122.5	C13—C12—H12	121.6
C14—C15—C16	106.1 (19)	C12—C13—N3	128.7 (5)
C14—C15—H15	126.9	C12—C13—C8	121.5 (5)
C16—C15—H15	126.9	N3—C13—C8	109.7 (4)
C17—C16—C15	115.3 (18)	C16'—C17—C16	112.4 (14)
C17—C16—H16	122.3	C16'—C17—C18	121.4 (11)
C15—C16—H16	122.3	C16—C17—C18	126.0 (10)
C17—S2'—C14'	93.3 (8)	C16'—C17—S2'	110.9 (11)
C15'—C14'—S2'	112.7 (15)	C18—C17—S2'	127.6 (4)
C15'—C14'—H14'	123.6	C16—C17—S2	110.6 (10)
S2'—C14'—H14'	123.6	C18—C17—S2	123.1 (5)
C14'—C15'—C16'	107 (2)	S2'—C17—S2	109.3 (4)
C14'—C15'—H15'	126.3	O3—C18—N4	126.4 (4)
C16'—C15'—H15'	126.3	O3—C18—C17	118.7 (5)
C17—C16'—C15'	115.7 (19)	N4—C18—C17	114.9 (5)
C17—C16'—H16'	122.1	O4—C19—N6	128.6 (5)
C15'—C16'—H16'	122.1	O4—C19—C20	125.8 (4)
C5—O1—Zn1	110.2 (3)	N6—C19—C20	105.6 (4)
C6—O2—Zn1	104.5 (3)	N5—C20—C21	137.5 (4)
C18—O3—Zn1	110.5 (3)	N5—C20—C19	114.7 (4)
C19—O4—Zn1	103.2 (3)	C21—C20—C19	107.8 (4)
H1W1—O1W—H1W2	108.7 (19)	C22—C21—C26	120.6 (5)
N2—N1—C5	108.4 (4)	C22—C21—C20	133.6 (5)
C7—N2—N1	120.2 (4)	C26—C21—C20	105.8 (4)
C7—N2—Zn1	119.8 (4)	C21—C22—C23	117.7 (6)
N1—N2—Zn1	120.0 (3)	C21—C22—H22	121.1
C6—N3—C13	111.3 (4)	C23—C22—H22	121.1
C6—N3—H3N	133 (4)	C22—C23—C24	121.1 (6)
C13—N3—H3N	116 (4)	C22—C23—H23	119.4
N5—N4—C18	108.0 (4)	C24—C23—H23	119.4
C20—N5—N4	121.4 (4)	C23—C24—C25	122.3 (6)
C20—N5—Zn1	120.7 (3)	C23—C24—H24	118.8
N4—N5—Zn1	117.8 (3)	C25—C24—H24	118.8
C19—N6—C26	111.1 (4)	C26—C25—C24	116.0 (6)
C19—N6—H6N	117 (4)	C26—C25—H25	122.0
C26—N6—H6N	131 (4)	C24—C25—H25	122.0
C28—N7—C27	115.1 (8)	C25—C26—C21	122.2 (6)
C28—N7—C29	127.0 (10)	C25—C26—N6	128.1 (5)
C27—N7—C29	117.7 (9)	C21—C26—N6	109.7 (4)
C2—C1—S1	112.2 (5)	O5—C27—N7	117.4 (10)
C2—C1—H1	123.9	O5—C27—H27	121.3
S1—C1—H1	123.9	N7—C27—H27	121.3
C1—C2—C3	113.9 (6)	N7—C28—H28A	109.5
C1—C2—H2	123.1	N7—C28—H28B	109.5
C3—C2—H2	123.1	H28A—C28—H28B	109.5
C4—C3—C2	111.7 (5)	N7—C28—H28C	109.5
C4—C3—H3B	124.1	H28A—C28—H28C	109.5
C2—C3—H3B	124.1	H28B—C28—H28C	109.5
C3—C4—C5	129.6 (5)	N7—C29—H29A	109.5
C3—C4—S1	110.8 (4)	N7—C29—H29B	109.5
C5—C4—S1	119.6 (4)	H29A—C29—H29B	109.5
O1—C5—N1	124.8 (5)	N7—C29—H29C	109.5
O1—C5—C4	120.5 (4)	H29A—C29—H29C	109.5
N1—C5—C4	114.7 (5)	H29B—C29—H29C	109.5
O2—C6—N3	129.0 (5)		

### Hydrogen-bond geometry (Å, °)

**Table:**

*D*—H···*A*	*D*—H	H···*A*	*D*···*A*	*D*—H···*A*
O1W—H1w1···O2	0.84 (1)	2.06 (3)	2.859 (5)	158 (6)
O1W—H1w2···O5	0.84 (1)	2.31 (8)	2.771 (9)	115 (7)
N3—H3N···O1W^i^	0.84 (1)	1.98 (1)	2.813 (6)	173 (5)
N6—H6N···O4^ii^	0.84 (1)	2.06 (2)	2.884 (5)	169 (5)

Symmetry codes: (i) −*x*, −*y*, −*z*+1; (ii) −*x*+1, −*y*+1, −*z*+1.

## References

[bb1] Barbour, L. J. (2001). *J. Supramol. Chem.* **1**, 189–191.

[bb2] Bruker (2001). *SMART* and *SAINT* Bruker AXS Inc., Madison, Wisconsin, USA.

[bb3] Rodríguez-Argüelles, M. C., Cao, R., García-Deibe, A. M., Pelizzi, C., Sanmartín-Matalobos, J. & Zani, F. (2009). *Polyhedron*, **28**, 2187–2195.

[bb4] Sheldrick, G. M. (1996). *SADABS* University of Göttingen, Germany.

[bb5] Sheldrick, G. M. (2008). *Acta Cryst.* A**64**, 112–122. 10.1107/S0108767307043930 18156677

[bb6] Westrip, S. P. (2010). *J. Appl. Cryst.* **43**, 920–925.

